# Case report: Management of pregnancy-associated immune thrombocytopenia in a French bulldog with dystocia

**DOI:** 10.3389/fvets.2024.1404337

**Published:** 2024-07-12

**Authors:** Rose Feldman, Kiko Bracker, Megan Whelan

**Affiliations:** MSPCA-Angell Animal Medical Center, Boston, MA, United States

**Keywords:** whelping, immune-mediated, immunology, ITP (idiopathic thrombocytopenic purpura), canine, brachycephalic, thrombocytopenia

## Abstract

**Introduction:**

The objective of this case report is to describe diagnosis and management of life-threatening immune thrombocytopenia (ITP) secondary to pregnancy in a dog with concurrent dystocia.

**Case summary:**

A 1-year 11-month old female intact French bulldog was referred for management of severe thrombocytopenia and spontaneous hemorrhage during whelping. The thrombocytopenia was progressive from approximately 32 days of gestation. In the absence of an identifiable cause for the thrombocytopenia, the patient was treated for ITP with immunosuppressive therapies and blood and plasma transfusions. The patient was also supported through dystocia until the platelet count normalized so a Caesarean section and ovariohysterectomy (OVH) could be performed.

**Discussion:**

This is the first report documenting ITP in a whelping canine. Pregnancy is a known trigger and can affect the clinical course of autoimmune diseases in women, including ITP. It is suspected that this patient’s pregnancy triggered ITP, paralleling what occurs in women.

## Introduction

Immune thrombocytopenia (ITP) is a common cause of severe thrombocytopenia in dogs and is a diagnosis of exclusion after eliminating primary causes of thrombocytopenia (including platelet consumption and decreased platelet production), and secondary causes of thrombocytopenia (including infectious disease, drug administration, and neoplasia) (1, 2). In people, ITP is an uncommon cause of thrombocytopenia, diagnosed after other etiologies have been excluded, and occurs in approximately 3.3 per 100,000 adults and 1.9–6.4 per 100,000 children per year. Patients generally present with a new onset of petechiae, purpura and mucosal bleeding at the time of diagnosis, with platelet counts frequently <20 × 10^9^/L. Despite the marked thrombocytopenia, few patients present with severe life-threatening bleeding (3). The link between autoimmune disease and pregnancy in women is well-studied, as both pregnancy and autoimmunity are modulated by T cell mediated-responses and sex hormones (4). Thrombocytopenia, defined as a platelet count of less than 150,000 platelets/μL, during pregnancy is well-documented in women. It occurs in up to 5–10% of pregnancies, with ITP making up approximately 3–4% of these cases (5, 6). Documented cases of autoimmune diseases associated with pregnancy in dogs include immune-mediated hemolytic anemia, immune-mediated polyarthropathy, and meningoencephalitis of unknown origin (7–9).

Despite the frequency with which it occurs in women, to the authors’ knowledge, this is the first reported case of ITP associated with pregnancy in a canine. This report describes management of a dog with ITP during dystocia.

## Case description

A 1-year 11-month old female intact nulliparous French bulldog, weighing 12.7 kg, presented to her primary veterinarian for routine prenatal evaluation at approximately 32 days of gestation after a natural breeding to an unknown sire. She was up-to-date on routine vaccinations, had no recent travel history, and was fed a commercial adult dog food. Physical examination was unremarkable. An ultrasound revealed possibly 4 fetuses without any apparent abnormalities. A fecal ova and antigen test showed moderate *Giardia* spp. cysts (11–30 per gram feces) and the remaining infectious disease testing including SNAP 4Dx Plus Test (IDEXX) and *Brucella canis* antibody via IFAT were negative. Comprehensive blood count (CBC) showed moderate thrombocytopenia [automated platelet count of 46 × 10^3^/μL; reference interval (RI): 143–448 × 10^3^/μL, manual count 30–50 × 10^3^/μL]. The serum chemistry profile was relatively unremarkable. The patient was treated with fenbendazole [1 teaspoon, *per os* (PO), every 24 h] for 5 days, but otherwise received no regular anti-parasitic prophylaxis or other medications.

Three days later (35 days gestation), a repeat CBC revealed a leukocytosis (25.2 × 10^3^/μL; RI: 4.9–17.6 × 10^3^/μL) characterized by neutrophilia (19.4 × 10^3^/μL; RI: 2.9–12.7 × 10^3^/μL) and static thrombocytopenia on manual count. The patient was prescribed amoxicillin/clavulanate potassium (20 mg/kg, PO, every 12 h) for presumptive infection.

Eight days later (43 days gestation), a CBC showed a resolved leukocytosis, normalized neutrophil count, new reticulocytosis (207 × 10^3^/μL; RI: 10–110 × 10^3^/μL) and persistent mild thrombocytopenia (manual count 50–100 × 10^3^/μL).

On day 57 gestation, the patient represented to the primary veterinarian for a scheduled abdominal radiograph with petechiation around the nipples. The patient was referred to the emergency service.

**Figure 1:**
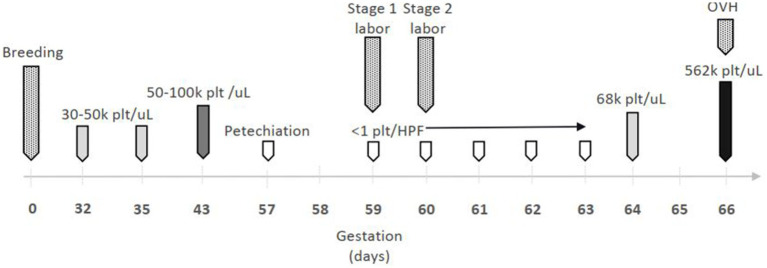
Timeline of gestational events and platelet (plt) count. OVH = ovariohysterectomy; 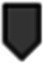 = mild thrombocytopenia; 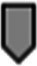 = moderate thrombocytopenia; 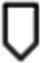 = severe thrombocytopenia; 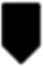 = thrombocytosis; 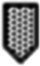 = gestational event.

On day 59 gestation, the patient was presented to the emergency service for evaluation of swollen, bruised nipples and signs of stage one labor (restlessness, panting, and hiding). On presentation, vital signs were within normal limits temperature of 100.0°F, heart rate (HR) of 120 beats per minute (bpm), and respiratory rate of 20 breaths per minute (brpm), her vulva and mammary glands were enlarged, and there was diffuse petechiation along her inguinal region and nipples. She was admitted to the hospital for management of presumptive ITP and possible dystocia. The patient’s platelet count and gestational events over time are shown in Figure 1. The owner prioritized the well-being of the patient over the unborn puppies and wanted to minimize risk to the patient in all case management decisions.

A venous blood gas analysis was relatively unremarkable. Relevant CBC and serum chemistry profile results included severe thrombocytopenia [2 × 10^3^/μL automated, <1 platelet seen per high-power field (HPF)], moderate hypochromic regenerative anemia [hematocrit (HCT) 23.8%, RI: 39.9–58.2%; mean corpuscular hemoglobin concentration (MCHC) 32.4 g/dL, RI: 33.8–37.5 g/dL; reticulocytosis of 318.7 × 10^3^/μL, RI: 13.8–125.1], and hypoalbuminemia (2.8 g/dL; RI: 3.1–4.2 g/dL). Blood type was DEA1 positive. An abdominal radiograph showed 4 fetuses; one was in anterior longitudinal presentation with the head positioned in the pelvic canal and was suspected to be able to pass. A point of care ultrasound (POCUS) showed at least 1 fetus with no heartbeat, 1 with a heart rate of 120 bpm, and 1 with heart rate of 200 bpm.

The following treatments were initiated for medical management of presumptive ITP: lactated Ringer’s solution (60 mL/kg/day, IV, CRI), dexamethasone sodium phosphate [0.28 mg/kg, intravenously (IV), every 24 h], maropitant (1 mg/kg, IV, every 24 h), and esomeprazole (1 mg/kg, IV, every 12 h) in anticipation of gastrointestinal bleeding secondary to thrombocytopenia. The patient began regurgitating and metoclopramide (2 mg/kg/day, IV, CRI) was started.

The next day (day 60 gestation), the patient developed progressive petechiation along the ventral abdomen and black discharge from her vulva. Vital signs remained within normal limits for the next 5 days. Ampicillin-sulbactam (50 mg/kg, IV, every 6 h) was started for possible bacterial metritis. A packed cell volume (PCV) and total solids (TS) revealed progressive anemia (18.5%, 5.8 g/dL respectively) and a 10 mL/kg DEA1 positive packed red blood cell (pRBC) transfusion was administered over 4 h. The patient continued to regurgitate with blood-tinged fluid and developed uterine contractions. Additional treatments included: methadone (0.1 mg/kg, IV, every 6 h), gabapentin (8 mg/kg, PO, every 8 h) for analgesia, and ondansetron (1 mg/kg, IV, every 6 h). The patient developed stranguria; a 10 French foley urinary catheter was placed and yielded marked hematuria. Administration of terbutaline (0.06 mg/kg, PO, every 8 h) was attempted to mitigate uterine contractions with limited success due to continued regurgitation. The post-transfusion PCV/TS was 27%/5.7 g/dL. The patient developed melena and passed one stillborn puppy overnight, after which contractions diminished.

The following morning (day 3 hospitalization, day 61 gestation), a CBC showed a regenerative macrocytic hypochromic anemia [HCT 16.9%; mean corpuscular volume (MCV) 74.1 fL, RI: 63.8–72.9; MCHC 32.0 g/dL; reticulocytes 266.1 × 10^3^/μL] and static thrombocytopenia with <1 platelet/HPF. A review of the blood spear performed by a board-certified pathologist showed no hemoparasites or major RBC abnormalities including spherocytes, ghost cells, or agglutination. A spun PCV/TS was 18%/4.2 g/dL and a second pRBC transfusion (10 mL/kg, IV, over 4 h) was given. Vincristine was administered (0.02 mg/kg, IV). Famotidine CRI (8 mg/kg/day, IV) and sucralfate suspension (40 mg/kg, PO, every 8 h) was initiated for continued melena and regurgitation. Mycophenolate mofetil (8 mg/kg, IV, every 12 h) was started as a second immunosuppressive agent. The post-transfusion PCV/TS was 22%/4.4 g/dL. She passed a second deceased fetus with few contractions.

On day 4 hospitalization (day 62 gestation) the patient had near-constant melena, mild petechiation over gums, and moderate to severe diffuse vulvar bruising. Another 10 mL/kg pRBC and 20 mL/kg fresh frozen plasma transfusions were administered. POCUS revealed a suspect hematoma in the bladder, and 2 fetuses with heart rates of 120 to 140 bpm. The urinary catheter was replaced due to functional obstruction and tranexamic acid (10 mg/kg, IV, every 6 h) was initiated. The following day (day 5 hospitalization), the PCV/TS was 11%/4.1 g/dL, <1 platelet/HPF on blood smear, and the patient was administered her fourth 10 mL/kg pRBC transfusion with a post-transfusion PCV/TS of 17%/4.4 g/dL. A final transfusion of cross-matched compatible freshly-donated whole DEA1 positive blood (20 mL/kg, IV, over 4 h) was administered.

On day 6 hospitalization (day 64 gestation), PCV/TS was 30%/6.9 g/dL with improved thrombocytopenia (68 × 10^3^/μL automated). Terbutaline was discontinued due to patient demeanor with administration of oral medications. The following day, the melena and hematuria improved; famotidine and tranexamic acid were discontinued. Uterine contractions resumed and she passed a third deceased fetus after a single dose of oxytocin [0.2 U/kg, intramuscularly (IM)].

On day 8 hospitalization (day 66 gestation), a CBC showed mild anemia (HCT 32.3%) and thrombocytosis (562 × 10^3^/μL). Caesarean section followed by OVH was performed routinely with one remaining deceased fetus found in the uterine body; a total of 4 stillborn fetus were delivered. She developed a fever of 103.4°F, increased respiratory effort with a rate of 40 brpm, and HR of 144 bpm post-operatively secondary to aspiration pneumonia. Her vital signs normalized with the addition of enrofloxacin (10 mg/kg, IV, every 24 h). The patient was discharged with prednisone (2 mg/kg, PO every 24 h), mycophenolate mofetil (10 mg/kg, PO, every 12 h), enrofloxacin (10 mg/kg, PO, every 24 h), maropitant (2 mg/kg, PO, every 24 h PRN), gabapentin (10 mg/kg, PO, every 8 h PRN), and Visbiome probiotics (1 capsule, PO, every 12 h).

One month later, the patient was clinically well with a normal platelet count. The prednisone was tapered over 3 weeks as it was thought that the inciting cause (pregnancy) had been resolved. However, she had a relapse of thrombocytopenia approximately 1 week after the last dose of prednisone, which was then restarted at its initial dosage.

Two weeks later, the patient had a thrombocytosis (842 × 10^3^/μL) with clumping. The prednisone was tapered over 3 weeks to approximately 0.36 mg/kg daily and the mycophenolate mofetil was continued at the same dose. One week after the steroid taper, a CBC was normal and the patient was clinically well.

## Discussion

This is the first case report describing diagnosis and treatment of ITP in a whelping canine. This case of ITP is suspected to be associated with pregnancy due to a lack of other causes of thrombocytopenia and response to immunosuppressive therapy.

The diagnosis of non-associative or primary ITP is dependent on excluding infection, neoplasia, drug administration, and consumptive coagulopathies, and relies on response to treatment with immunosuppressive therapies that can include corticosteroids, other immunosuppressive drugs, and human immunoglobulin (1, 10). According to the recently published guidelines for ITP, cytologic evaluation of the bone marrow is not recommended as a routine diagnostic assay in dogs with non-associative/primary ITP and platelet/megakaryocyte-associated antibody tests are not diagnostic for primary ITP alone so were not performed in this case (2, 11, 12).

The pathophysiology of ITP in dogs is incompletely understood and largely extrapolated from human literature. A few established mechanisms of disease include: autoantibodies targeting platelet surface glycoproteins which are cleared from the mononuclear phagocytic system; pro-inflammatory T helper cell (Th1, Th17 and Th22) cytokines promoting macrophage function, autoreactive B cell development, and T cell cytotoxicity; dysfunctional T and B regulatory cells responsible for self-tolerance; and inappropriately low thrombopoeitin (TPO) levels (13). Pregnancy is a state of immunomodulation related to T cell-mediated responses towards the conceptus—considered a semi-allograft—that prevent rejection and allow tolerance for the fetus to be carried to term (4). Progesterone, the predominant hormone of pregnancy, has been shown to have immunomodulatory effects, including directly on CD4^+^ T helper cells (14).

ITP is the most common cause of low platelets in the first and second trimesters of pregnancy in women (5). Approximately one-third of women with ITP are diagnosed during pregnancy, and about half of women with previously diagnosed ITP have a significant platelet decline during pregnancy (5, 15). While hemorrhage from vaginal delivery is unusual with platelet counts greater than 50 × 10^9^/L, the aim of treatment is to achieve platelet counts greater than or equal to 80 × 10^9^/L; treatment is only recommended in the third trimester if there is a platelet count less than 30 × 10^9^/L, bleeding, or a planned procedure to avoid risks to the mother and exposure to the fetus (4, 16). Corticosteroids are the first-line therapies in humans with ITP requiring treatment. Second-line therapies are indicated for adults who are corticosteroid-dependent or unresponsive to corticosteroids and include either treatment with a TPO receptor antagonist, rituximab, or splenectomy. Other immunomodulatory treatments, considered third-line therapies, include azathioprine, cyclophosphamide, cyclosporine A, danazol, dapsone, mycophenolate mofetil, and vinca alkaloids; only azathioprine is considered “safe” for use during pregnancy and lactation (17, 18). Platelet transfusion during delivery is used in 5–18.9% of patients (16).

In dogs, initial therapies for treatment of ITP include corticosteroids, secondary immunomodulatory drugs (e.g., azathioprine, cyclosporine, leflunomide, and mycophenolate mofetil), vincristine, hIVIG, and splenectomy (10). In this case, corticosteroids and vincristine were used as well as the second-line therapy mycophenolate mofetil. Vincristine in addition to glucocorticoids has been shown to significantly reduce time to platelet recovery compared with glucocorticoids alone (19), and is now recommended as a first line emergency adjunctive treatment for dogs with ITP and clinically relevant bleeding (2). A more recent study comparing vincristine and hIVIG as a second agent showed no significant difference in time to platelet recovery (20). While one study found that a single infusion of hIVIG within 24 h of initiation of corticosteroid therapy significantly reduced platelet recovery time and duration of hospitalization, another recent study examining the use of hIVIG as a salvage treatment for ITP cases did not show clinical benefit in survival rate (21, 22). Mycophenolate mofetil was chosen over the other options due to ease of dosing, perceived reduced gastrointestinal side effects over the other agents, and cost. The time to platelet recovery in this case was 6 days, which was likely affected by the multiple sources of bleeding and delay in vincristine administration as a result of difficulty obtaining fresh IV access.

A limitation of this case report was the lack of coagulation testing. Ideally, viscoelastic testing would have been performed to evaluate the theoretical benefit of treatment with the antifibrinolytic tranexamic acid (TXA) in the face of her urinary obstruction with a bladder hematoma. However, all blood sampling was performed using a peripherally inserted size 4Fr 30 cm central line catheter due to limited venous access, and there was no literature available evaluating the accuracy of viscoelastic testing using the VCM-Vet with blood drawn in this manner. While a 2021 meta-analysis of human patients with GI bleeding treated with low-dose (less than 2 g IV over 24 h) TXA was suggestive of lessened transfusion requirements (23), a more recent study on the use of TXA in 4 dogs with ITP was not suggestive of a clinical benefit (24). Other coagulation testing was not performed due to financial limitations of the owner.

The patient’s concurrent dystocia was managed by attempting to ameliorate signs of labor versus immediate platelet transfusion and Caesarean section because the owner was unwilling to put the patient’s life at unnecessary risk. More aggressive medical management of dystocia using oxytocin was not initially performed given the brachycephalic breeding, though the puppies that passed vaginally did so without obstruction. While there is no literature available on the use of terbutaline to prevent contractions during at-term whelping, a combination of intravenous, subcutaneous, and oral terbutaline has been shown to arrest premature labor in women (25, 26). As the patient’s safety was prioritized over that of the unborn puppies, vincristine was administered despite the risks of teratogenicity. However, favorable pregnancy outcomes have been reported with the administration of vincristine in the second and third trimesters in women (12), with no literature available on its effects in pregnant or whelping bitches.

While it is not possible to prove that this patient’s ITP was caused by pregnancy, the greater incidence of autoimmune disease in women (14) and female dogs (1), the typical effects of pregnancy on immune-mediated disease including ITP, and the other case reports of immune-mediated disease during pregnancy in dogs (8–7), it is likely that this patient’s thrombocytopenia was worsened, if not caused, by her pregnancy, and her treatment was complicated by dystocia.

This is the first reported case of ITP in a pregnant canine and adds to a growing body of evidence that suggests canines are susceptible to the same immunomodulatory effects of pregnancy as women.

## Data availability statement

The raw data supporting the conclusions of this article will be made available by the authors, without undue reservation.

## Ethics statement

Ethical approval was not required for the studies involving animals in accordance with the local legislation and institutional requirements because this is a case report of an animal treated at MSPCA-Angell Animal Medical Center. Written informed consent was obtained from the owners for the participation of their animals in this study. Written informed consent was obtained from the owners of the animals for the publication of this case report.

## Author contributions

RF: Investigation, Writing – original draft, Writing – review & editing. KB: Writing – review & editing. MW: Writing – review & editing.
